# Circulating levels of inflammatory cytokines and angiogenesis-related growth factors in patients with osteoarthritis after COVID-19

**DOI:** 10.3389/fmed.2023.1168487

**Published:** 2023-07-06

**Authors:** Daryna Krenytska, Kateryna Strubchevska, Marko Kozyk, Tetiana Vovk, Tetiana Halenova, Larysa Kot, Nataliia Raksha, Olexii Savchuk, Tetyana Falalyeyeva, Olena Tsyryuk, Liudmyla Ostapchenko

**Affiliations:** ^1^Institute of Biology and Medicine, Taras Shevchenko National University of Kyiv, Kyiv, Ukraine; ^2^William Beaumont Hospital, Royal Oak, MI, United States

**Keywords:** osteoarthritis, COVID-19, inflammation, cytokines, angiogenesis, growth factors, hypoxia-inducible factors, heat shock proteins

## Abstract

**Background:**

The disease COVID-19, caused by SARS-CoV-2 infection, has a systemic effect and is associated with a number of pathophysiological mechanisms that mobilize a wide range of biomolecules. Cytokines and growth factors (GFs) are critical regulators of tissue damage or repair in osteoarthritis (OA) and are being recognized as key players in the pathogenesis of COVID-19. A clear understanding of the long-term consequences of SARS-CoV-2 infection, especially in patients with concomitant chronic diseases, is limited and needs to be elucidated. The study aimed to evaluate the degree of inflammation and levels of pro-angiogenic and hypoxic factors, as well as heat shock proteins HSP60 and HSP70 in plasma, of patients with OA after recovery from COVID-19.

**Methods:**

The research involved patients of an orthopedic specialty clinic aged 39 to 80 diagnosed with knee OA. All examined patients were divided into three groups: the Control group included conditionally healthy donors, group OA included patients with knee OA mainly stage II or III and the group of OA and COVID-19 included patients with OA who had COVID-19. The plasma levels of pro-inflammatory molecules IL-1β, IL-6, TNF-α, NF-κB, angiogenic factors VEGF, FGF-2, PDGF, hypoxic factor HIF-1α and molecular chaperones HSP60 and HSP70 were measured by enzyme-linked immunosorbent assay.

**Results:**

The study showed that in both groups of patients, with OA and convalescent COVID-19, there was an increase in the plasma level of IL-1β and a decrease in TNF-α and NF-κB levels when compared to healthy controls. Systemic deregulation of the cytokine profile was accompanied by reduction in plasma levels of pro-angiogenic growth factors, most pronounced in cases of VEGF and PDGF. This analysis did not reveal any significant difference in the plasma level of HIF-1α. A decrease in the level of stress protein HSP60 in the blood of patients with OA, as well as those patients who have had SARS-CoV-2 infection, has been established.

**Conclusion:**

The results suggest the potential role pro-inflammatory cytokines and angiogenesis-related growth factors in pathogenesis of both joint pathologies and long-term systemic post-COVID-19 disorders.

## Introduction

Coronavirus disease 2019 (COVID-19), caused by severe acute respiratory syndrome coronavirus 2 (SARS-CoV-2) was first detected in December 2019 in Wuhan, China, which quickly escalated into a global worldwide pandemic that continues to affect all aspects of people’s daily lives (1). Although COVID-19 is primarily associated with respiratory symptoms, numerous studies have reported various extrapulmonary manifestations of the coronavirus infection and its deleterious effects on almost all organism systems (2). As is known, in patients (even after recovery) structural and functional disorders of the respiratory, cardiovascular, nervous, digestive and musculoskeletal systems, have been observed (3, 4). Joint pain, myalgia, and neuropathy/myopathy are typical signs of COVID-19, however, their prevalence has not yet been systematically studied (5). Approximately 4 to 12% of patients still have joint pain during the first year after infection (6). The long-term effects of COVID-19 including the musculoskeletal system are just beginning to be assessed. Among the probable reasons for the development of complications being considered are the persistence of the pathogen, oxygen deficiency, a prolonged state of hyperinflammation, autoimmune processes, individual features of the patient’s immune response, endothelial dysfunction, vitamin D deficiency, fibromyalgia and disorders in the hemostasis system (7–10). It has been previously established that COVID-19 is associated with the worsening symptoms of osteoarthritis (OA), but there is insufficient evidence that the SARS-CoV-2 triggers the development of this joint pathology (11). OA is one of the most common diseases in the elderly, who, along with individuals with chronic pathologies such as diabetes, cardiovascular or pulmonary diseases, are at a greater risk of infection with SARS-CoV-2, high-severity COVID-19, and the occurrence of post-COVID-19 complications. The progression of OA leads to inflammation of all articular structures, cartilage degradation, subchondral bone remodeling, hypertrophy of the joint capsule, osteophytes formation, and muscle atrophy, which is the main cause of joint pain, functional constraint and disability. Currently, research of the biochemical mechanisms of the potential impact of SARS-CoV-2 infection on degenerative processes in cartilage or the development of inflammation in joints is still at an initial stage (12). Previous studies have shown that coronavirus infection in patients causes a pro-inflammatory state with systemic effects that may play an important role in the development of joint and bone pathology (5, 11). Scientific sources suggest that the inflammatory reaction in the organism, even of a low degree, can generate a large number of circulating pro-inflammatory cytokines, including tumor necrosis factor (TNF)-α, interleukins (IL), chemokines, matrix components and proteases. These regulatory molecules have numerous mechanisms by which they can cause the destruction of cartilage, thus the inducing of OA or worsening its course (13, 14). The inflammatory effects of key cytokines such as IL-1β, IL-6 and TNF-α are realized through the appropriate intracellular signaling pathways resulting in the activation of nuclear factor kappa B (NF-κB), JAK/STAT and MAPK pathways (15). Inflammation with the release of pro-inflammatory mediators and oxidative stress, in turn, can directly or indirectly stimulate the cascade of angiogenic process such as the formation of new blood vessels from their pre-existing microvascular structures, which could facilitate the invasion of inflammatory cells that is important in the pathogenesis of inflammatory diseases of the joints, in particular OA. Angiogenesis and inflammation are interdependent processes. Chronic inflammation caused by the long-term pathological process in the joint and persistence of the SARS-CoV-2 virus is almost always accompanied by angiogenesis, but angiogenesis can also occur in the absence of inflammation. Angiogenesis is a precisely regulated process and requires coordinated signaling events, involving various growth factors (GFs), primarily vascular endothelial growth factor (VEGF), fibroblast growth factor (FGF), and platelet-derived growth factor (PDGF). A powerful stimulator of angiogenesis is tissue hypoxia, which often occurs in inflamed tissues, and activation of key regulators of the cellular response to decreases in available oxygen—hypoxia-inducible factors (HIFs). It is known that transcription factor HIF-1α plays an important role in articular cartilage (hypoxic tissue) since it has a protective effect in maintaining the cartilage matrix (16). An important role in maintaining cellular homeostasis in the pathogenesis of almost all diseases, including the musculoskeletal system, is played by heat shock proteins (HSPs) (17). As intracellular molecular chaperones, HSPs ensure proper folding, stabilization and translocation of both normal and damaged oligomeric proteins and are responsible for protecting cells from stress factors, presenting immune and inflammatory cytokines which are important factors in the regulation of cellular differentiation, survival and death (18). Their level increases in places of acute and chronic inflammation. HSPs have a wide range of molecular weights and, accordingly, can be classified into different groups such as small heat shock proteins, i.e., HSP21 and HSP10, medium, i.e., HSP60 and HSP70, and large heat shock proteins, i.e., HSP90 and HSP100. There are a large number of studies reporting that HSPs are not only involved in antiviral reactions, but can also be used by the virus both to fold its own proteins and to increase the chances of survival in adverse conditions inside the host cells. Current researches have shown the biological significance of HSPs in the course of SARS-CoV-2 infection (18). HSP60 has been shown to be involved in inappropriate inflammatory responses that exacerbated the progression of COVID-19 (19). While the overexpression of HSP70 in patients with COVID-19 indicates its importance in all viral processes, including cell penetration, viral replication, and virion assembly (20). Considering the above, the aim of this study was to evaluate the degree of inflammation and to determine the plasma levels of pro-angiogenic factors such as VEGF, FGF-2, PDGF and HIF-1α, as well as heat shock proteins HSP60 and HSP70 in patients with knee OA following COVID-19.

## Materials and methods

### Participants and study design

This study involved 36 patients (24 women and 12 men), aged 39–80 years, with an established history of knee OA, who were on out-patient treatment in the orthopedic specialty clinic (Medical Center Orthoclinic) in Ternopil city, Ukraine. At the selection stage, all patients with OA underwent X-ray examination of the knee joints in standard anteroposterior and lateral projections, in a standing position with no load on the examined joint when bending the knee by 30°. The stage of joint damage was determined using the Kellgren-Lawrence classification. To assess the function status of patients with knee OA, an index was calculated according to the WOMAC (Western Ontario and McMaster Universities) questionnaire. The examined patients were divided into the following groups: the first group—Control group (*n* = 10)—conditionally healthy donors without clinical signs of the musculoskeletal pathologies and who did not suffer from COVID-19 in the past; the second group—OA (*n* = 22)—patients with knee OA mainly stage II or III; the third group—OA and COVID-19—included 14 patients with OA of the knee joints who had mild to moderate COVID-19 6–9 months prior to examination. The presence of a history of SARS-CoV-2 infection (in the selection of 2nd research groups) or its absence (in the Control group and 1st groups patients with OA) was previously confirmed in the laboratory *via* real-time reverse transcription polymerase chain reaction (RT-PCR) assay using nasopharyngeal swabs sample and/or SARS-CoV-2 antibody testing (Table 1).

**Table 1:** Basic patient information.

Patient, ID	Gender	Age (years)	Stage of knee OA	COVID-19	Patient, ID	Gender	Age (years)	Stage of knee OA	COVID-19
1	Male	40	І	+	19	Male	42	ІІ	−
2	Female	63	ІV	−	20	Female	62	ІІІ	+
3	Female	61	III	−	21	Female	45	ІІ	−
4	Female	69	ІІІ	+	22	Female	52	ІІ	−
5	Female	49	ІІ	−	23	Male	67	ІІІ	+
6	Male	45	II	+	24	Male	68	ІІІ	−
7	Female	61	ІІ-ІІІ	−	25	Male	70	ІІІ	−
8	Female	66	ІІІ	−	26	Female	42	ІІ	−
9	Female	68	ІІІ	+	27	Female	68	ІV	+
10	Male	55	ІІІ	+	28	Female	63	ІІІ	+
11	Male	80	ІІ	−	29	Male	66	ІІІ	+
12	Male	56	ІІІ	+	30	Female	67	ІІІ	+
13	Female	65	ІІІ	−	31	Female	68	ІІІ	−
14	Female	48	ІІ	−	32	Female	51	ІІ	−
15	Male	78	ІІ	−	33	Female	41	ІІ	−
16	Male	39	ІІ	−	34	Female	67	ІII	+
17	Female	60	ІІ	−	35	Female	42	I	−
18	Female	66	ІІІ	−	36	Female	68	ІІІ	+

All the patients and healthy donors were informed in detail about the conduct of the clinical research and had given written informed consent to participate in the study. The study was performed in accordance with the principles outlined in the World Medical Association’s Declaration of Helsinki and fully met the ethical and moral requirements of the current provisions of the Ministry of Health of Ukraine and approved by the Ethics Committee at the ESC “Institute of Biology and Medicine,” Kyiv, Ukraine (the protocol No 3, 12.04.2022).

### Sample collection and processing

In the morning, venous whole blood was collected from each patient and healthy controls into commercially available citrate-treated tubes. All participants fasted for 8 h before blood sampling. Processing of the obtained blood samples included centrifugation at 3000 rpm in 10 min at 4°C to avoid platelets’ activation. Following centrifugation, the plasma supernatant was removed and immediately transferred into a clean polypropylene tube, quickly frozen at − 20°C, transported and stored, until assayed.

### ELISA analysis

The plasma levels of pro-inflammatory cytokines IL-1β, IL-6, TNF-α and transcription factor NF-κB; levels of angiogenesis-related growth factors VEGF, FGF-2, PDGF and HIF-1α; the heat shock proteins HSP60 and HSP70 levels, were measured by enzyme-linked immunosorbent assay (Commercial ELISA kits, Biotrak ELISA System, Healthcare, United States). All assay procedures were performed according to the manufacturer’s instructions. Control samples were analyzed simultaneously on each plate for every marker. In brief, the samples were incubated in 96-well plates during the 12 h at 4°C. Removal of unabsorbed antigen was washed three times wells with 50 mmoL/L Tris–HCl buffer (pH 7.4) containing 0.1% Tween-20. To block non-specific binding sites, a 5% solution of skimmed milk was used. Primary polyclonal antibodies (Santa Cruz Biotechnology, United States) was used, as were secondary antibodies conjugated with horseradish peroxidase (Bio-Rad, United States) and o-phenylenediamine (OPD) substrate in the presence of hydrogen peroxide (Sigma-Aldrich, Louis St, MO). Optical the density of the samples was measured on a microplate reader (“BioTek Instruments, Inc.,” United States) at wavelength 492 nm.

### Statistical analysis

Statistical analysis was performed using GraphPad Prism software version 9.4.0 (GraphPad Software Inc., United States). Testing the hypothesis of the normal distribution of samples was carried out using the Shapiro–Wilk criterion. Results between the groups were assessed by one-way analysis of variance (ANOVA) with Tukey’s multiple comparisons test. If the sample does not meet the criteria for normal distribution, the reliability of the differences between the samples was determined using the Kruskal–Wallis H test followed by the Dunn’s multiple comparison test. The results were presented as the mean ± standard deviation (SD) for each group. Differences were considered as statistically significant at a value of *p* ≤ 0.05.

## Results

**Figure 1:**
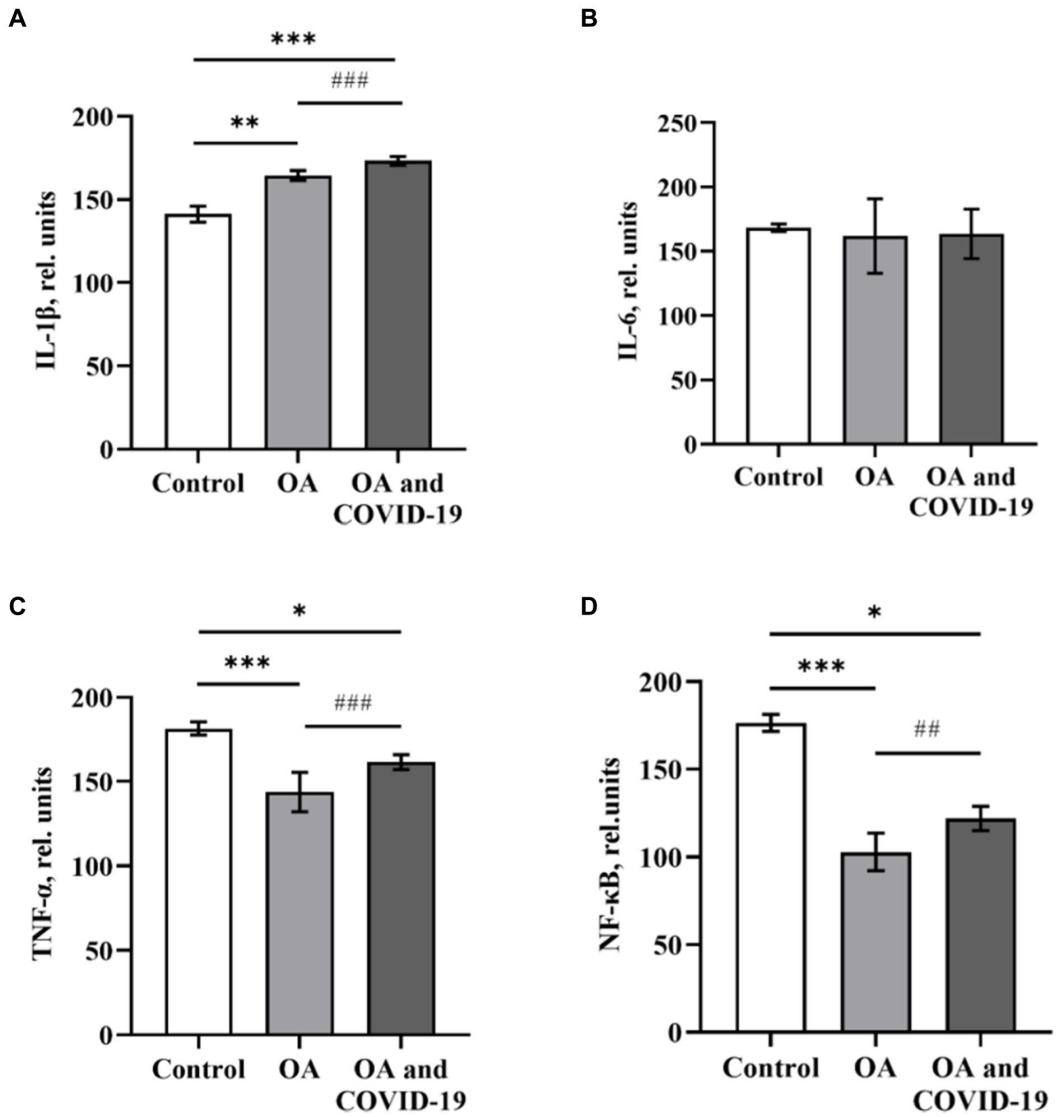
The levels of pro-inflammatory cytokines IL-1β **(A)**, IL-6 **(B)**, TNF-α **(C)** and transcription factor NF-κB **(D)** in plasma of patients with osteoarthritis (ОА, *n* = 22), after recovery from COVID-19 (ОА and COVID-19, *n* = 14), and healthy control (Control, *n* = 10), relative units. Data are presented as the mean ± SD. Statistical analysis based on the One-way ANOVA; ^***^p ≤ 0.001, ^**^p ≤ 0.01 and ^*^p ≤ 0.05, comparing to Control group; ^###^*p* ≤ 0.001, and ^##^*p* ≤ 0.01, comparing to group of patients with OA who have not had COVID-19.

Pro-inflammatory cytokines are secreted by various types of immune cells (monocytes, macrophages, NK cells, T and B cells), as well as fibroblasts, endothelial and epithelial cells in response to various states of organism, including the activation of the innate immune system. Evaluation of the cytokine profile in the blood, provides the opportunity to obtain information about the activation status of immunocompetent cells, the intensity of the inflammatory process at the system level, and also to predict the degree of development and severity of the disease. Analysis of the level of key pro-inflammatory mediators, which play a significant role in the catabolism of articular cartilage in the blood plasma of patients with OA, showed a statistically significant higher level of cytokine IL-1β by 16.4% and a decrease in the level of TNF-α—by 22.1%, compared to the values of the Control group [*p* ≤ 0.01 and *p* ≤ 0.001, respectively (Figures 1A,C)]. At the same time, the level of the IL-6 did not statistically differ from the level of healthy control (Figure 1B). Studies have found a decrease in the level of the transcription factor NF-κB by 41.7% compared to the control (*p* ≤ 0.001) in the plasma of patients with OA (Figure 1D).

The data show that in patients with OA, who have had COVID-19, changes in the plasma levels of pro-inflammatory cytokines were unidirectional with changes in such parameters in the absence of viral damage (Figure 1A–D). Patients with joint pathology, who recovered from SARS-CoV-2 infection, had an increase in plasma of IL-1β level by 22.7%, compared to control (p ≤ 0.001), and by 5.4%, comparing to group of patients with OA in the absence of a history of coronavirus disease (p ≤ 0.001; Figure 1A). The levels of TNF-α and NF-κB under similar conditions decreased by 11 and 30.8%, respectively, compared to healthy controls (p ≤ 0.05), but had a slight increase in both groups by 14.3 and 18.7%, respectively, compared to patients with OA, who did not suffer from COVID-19 (p ≤ 0.001 and p ≤ 0.01, respectively; Figures 1C, D).

**Figure 2:**
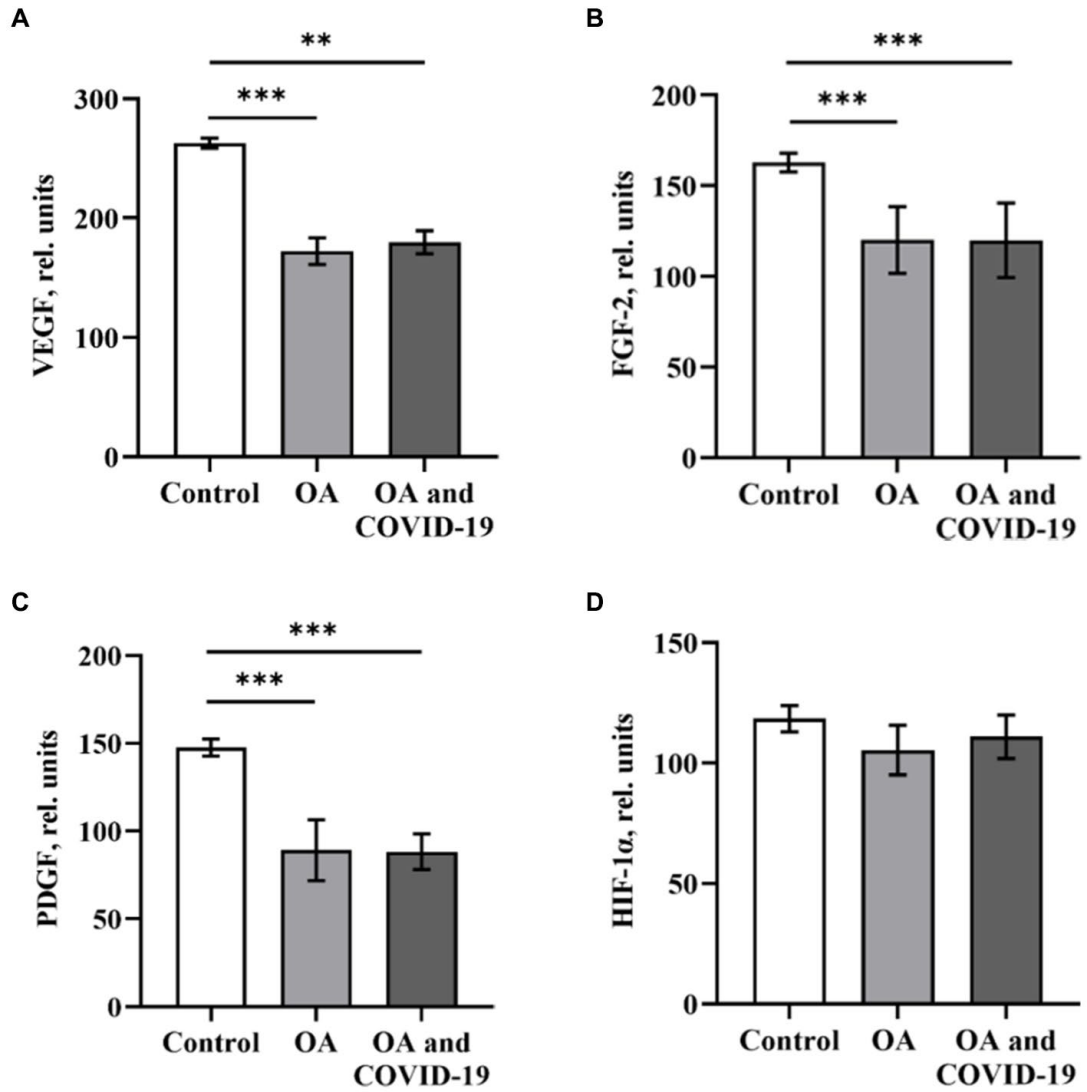
The levels of angiogenesis-related growth factors VEGF **(A)**, FGF-2 **(B)**, PDGF **(C)** and HIF-1α **(D)** in plasma of patients with osteoarthritis (ОА, *n* = 22), after recovery from COVID-19 (ОА and COVID-19, *n* = 14), and healthy control (*n* = 10), relative units. Data are presented as the mean ± SD. Statistical analysis based on the One-way ANOVA; ^***^p ≤ 0.001 and ^**^p ≤ 0.01, comparing to Control group.

We studied the profile of pro-angiogenic biomarkers in the plasma of patients with OA from 6 to 9 months after SARS-CoV-2 infection. Our research showed a significant reduction in VEGF, FGF-2 and PDGF levels in patients with OA, who had COVID-19 and those who did not have a history of SARS-CoV-2 infection. Thus, in patients with OA who did not suffer from COVID-19, established a decrease in the levels of VEGF by 34.3%, FGF-2 by 26.2% and PDGF by 39.7%, compared to healthy control (*p* ≤ 0.001). At the same time, in patients with OA who were infected with the coronavirus the values in the levels of VEGF, FGF-2 and PDGF were lower by 31.4, 26.3 and 40.2%, respectively, then the values of Controls group (*p* ≤ 0.01 and *p* ≤ 0.001, respectively; Figures 2A–C). This analysis did not reveal any significant difference in plasma levels of the above-mentioned angiogenesis-related GFs when comparing groups of patients with joint pathology in the post-COVID-19 period and the absence of coronavirus lesions.

**Figure 3:**
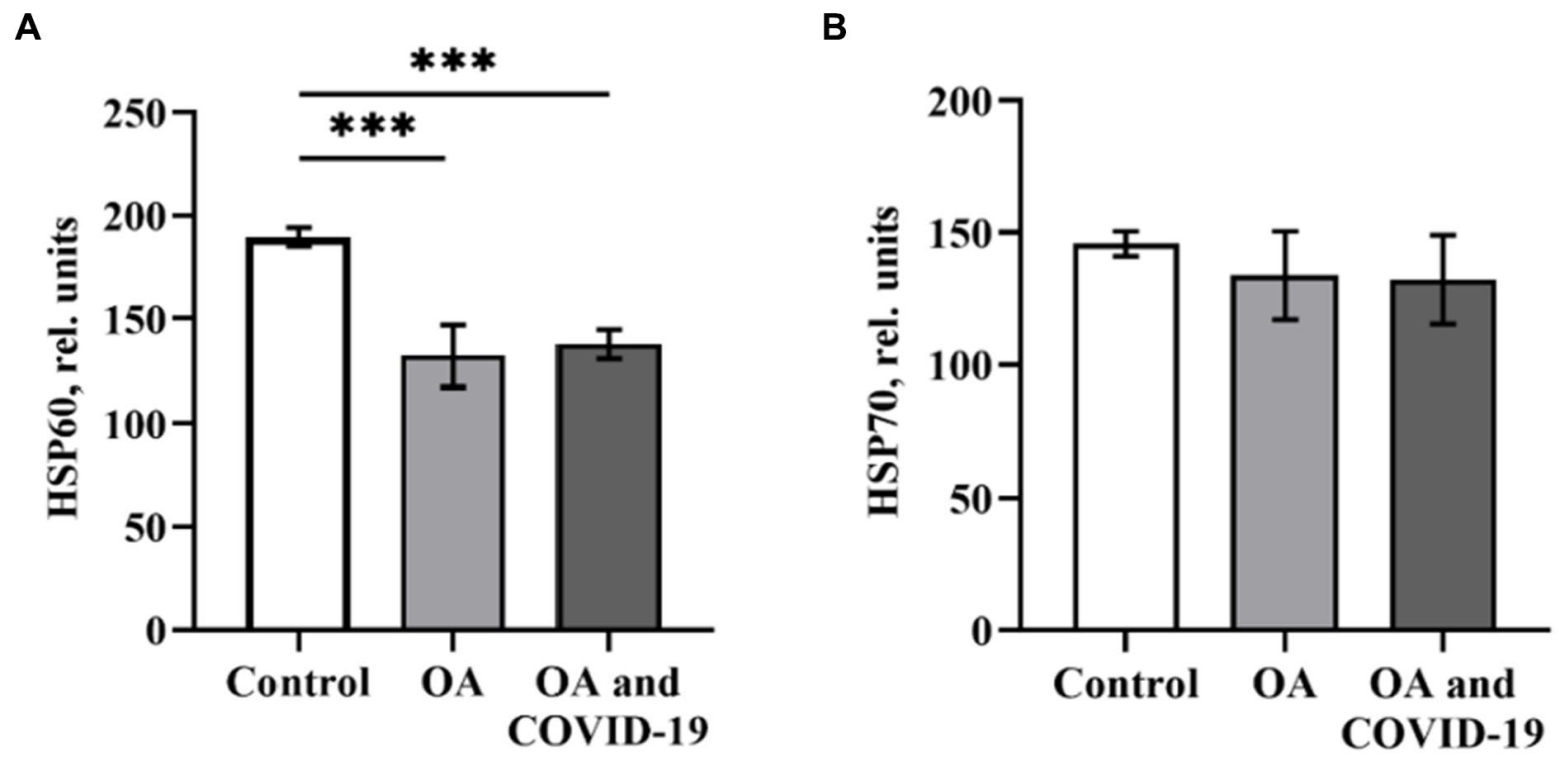
The level of heat shock proteins HSP60 **(A)** and HSP70 **(B)** in plasma of patients with osteoarthritis (ОА, *n* = 22) after recovery from COVID-19 (ОА and COVID-19, *n* = 14), and healthy control (*n* = 10), relative units. Data are presented as the mean ± SD. Statistical analysis based on the One-way ANOVA; ^***^p ≤ 0.001, comparing to Control group.

A critical role in the regulation of pro-angiogenic molecules, among which the most prominent regulator of both physiological and pathological angiogenesis is VEGF, is played by the transcription factor HIF-1α. Against the background of a decrease in the levels of the main angiogenic parameters in patients with OA in conditions of both the presence and absence of a history of SARS-CoV-2 infection, we have not established any reliable changes in the plasma levels of HIF-1α (Figure 2D). Many studies have demonstrated the role of HSPs, which can ensure the preservation of protein homeostasis in the organism, in attenuation of inflammation, and in reducing the severity of the disease. It has been shown (21) that HSP60 and HSP70 can affect the innate and adaptive immune response as well as the autoimmune reactions that reflect the state of cells and tissues in certain diseases, including OA. Viruses, in particular SARS-CoV-2, can induce high expression of chaperones as a cellular defense measure and use host HSPs to fold their own proteins, thus increasing infection efficiency (18). When determining the levels of heat shock proteins HSP60 and HSP70 in plasma of patients with OA, we found a decrease in the level of HSP60 by 30.3%, compared to healthy control (*p* ≤ 0.001; Figure 3A). The decreased level of HSP60 by 27.3%, when compared to control (*p* ≤ 0.001), also persisted in patients with joint pathology after a long post-COVID-19 period (Figure 3A). At the same time, the level of HSP70 in the blood plasma of OA patients who did not have COVID-19 and those who were infected with SARS-CoV-2 was within the range of values of the Control group (Figure 3B).

## Discussion

Novel coronavirus disease 2019, which is associated with a variety of clinical manifestations ranging from mild or asymptomatic to acute respiratory distress syndrome and multiorgan failure, is known to be related with multisystem disorder and may cause long-term consequences, especially in patients with concomitant chronic diseases (22). It is possible that the inflammation caused by COVID-19 negatively affects the musculoskeletal system through numerous proposed mechanisms. About 10% of patients who recovered had persistent symptoms of musculoskeletal origin, which included chronic fatigue, myalgia, swelling and joint pain, during the first year after primary infection (6). Researches have shown that SARS-CoV-2 infection causes a significant systemic increase in the level of pro-inflammatory cytokines, such as IL-1β, IL-6, IL-8, IL-17 and TNF-α, which are the main mediators participating in the pathogenesis of OA (23). They have the greatest effect on cartilage homeostasis and can induce catabolic and destructive processes in the joint. It should be noted that the increased level of inflammatory mediators promotes vasodilation and induces dysfunction of endothelial cell, which can result in impaired blood circulation in the synovial membrane and subchondral bone, degenerative changes in articular cartilage, stimulation of osteocyte apoptosis and osteoclast formation. The data obtained in the present study showed that patients with OA and those who recovered from COVID-19 6–9 months before had elevated plasma levels of IL-1β and decreased levels of TNF-α and NF-κB. IL-1β and TNF-α, produced by immune cells, especially mononuclear cells, as well as activated chondrocytes, synoviocytes, and osteoblasts, are among the mediators that are released mainly in the early stages of OA (24). They are known to control the inflammatory cascade, induce the synthesis of matrix metalloproteinases (MMPs), nitric oxide, prostaglandin E2 (PGE2) and IL-6, reduce the synthesis of the main components of the extracellular matrix (ECM), while inhibiting the anabolic activity of chondrocytes and reduce the production of type II collagen (25). Catabolic events involving IL-1β and TNF-α are mediated through the activation of NF-κB intracellular signaling cascades. It is shown that patients with OA had an elevated level of IL-1β in the synovial fluid, synovial membrane, cartilage, and in the subchondral bone layer, which could indicate local chronic inflammation in the knee joint and progression of OA (26, 27). The levels of IL-6 and TNF-α in the synovial fluid and serum of patients with OA vary depending on the stage of joint pathology (28). Fathi, et al., similar to our results, also showed an increase in the IL-1β level and a statistically significant decrease in the level of pro-inflammatory cytokines, such as IL-1α, IL-6 and TNF-α in patients during recovery from coronavirus disease (29). It is known that IL-1β plays a significant role in enhancing the inflammatory cascade, thus carrying out numerous biological effects such as inflammatory response, vasodilatation, angiogenesis, leukocyte recruitment, T-cell differentiation, etc. both in places of local inflammation, in particular in the joint, at a systemic level (30). Studies have found an increase in the circulatory IL-1β level at different stages of COVID-19, including in mild SARS-CoV-2 infection, but this cytokine had a late peak 2–3 months after infection (31). Ong et al. (32) reported that in patients, even 6 months after recovery from COVID-19, systemic cytokine profiles differed from conditionally healthy control: elevated levels of many GFs, cytokines, including IL-1β, and chemokines were maintained, regardless of the severity of the disease. We have established a more significant increase in the level of IL-1β in patients with OA in the post-COVID-19 period, comparing to patients in the absence of viral lesions, as well as other pro-inflammatory cytokines (32), associated with T cells, may be related with increased activation and proliferation of SARS-CoV-2 specific CD4 and CD8 T cell clonotypes, as well as reduction of NK cells, that is characteristic of convalescents for a long period of time after the disappearance of the infection (33). TNF-α is a pleiotropic cytokine that activates the immune system to regulate the normal immune response, but the biological effect of TNF-α as a pro-inflammatory mediator depends on its concentration in the tissues. It’s inappropriate or excessive production can be harmful and lead to the development of pathological conditions (34). TNF-α, by binding to the appropriate receptor, activates transcription factors through the NF-κB signaling pathway, causing an inflammatory response similar to that of causing IL-1β, but is nonetheless weaker (28). Decreased activity of the transcription factor NF-κB can suppress the release of cytokines, particularly TNF-α. It can be assumed that some effects of TNF-α can be blocked at a level below the receptors by inhibitors of NF-kB. Recovery from COVID-19 is characterized by a decrease in the number of lymphoid cells, changes in the molecular regulation of the immune system and cytokine profile. Since most pro-inflammatory mediators act mainly locally, their concentration in the synovial fluid under conditions of OA can be higher than in the blood, but in case of failure of local protective reactions, their action is manifested at the systemic level. Despite the fact that IL-6 is considered a particularly important cytokine in chronic inflammatory diseases and in the pathogenesis of COVID-19, it is still a predictor of its progression (35). In the present work, we did not establish a significant difference, compared to healthy controls in plasma the level of IL-6 of patients with OA, as well as in patients with joint pathology who were infected with SARS-CoV-2. The obtained data could indicate a weakening of the immunopathological manifestations of the viral disease and a relative remission of joint pathology. Similar results have been observed in recent studies (36), which indicate the absence of statistically significant differences in the serum concentration of IL-6 between patients of the control group and those recovered from the COVID-19. The systemic deregulation of cytokines in patients with OA as well as in those who have had SARS-CoV-2 infection, found in this study, is consistent with our previously obtained data on the imbalance of pro- and anti-inflammatory cytokines in the blood plasma of patients with joint pathology after a long post-COVID-19 period (37), and may indicate different immunoregulatory mechanisms involved in the pathogenesis of these diseases. The contribution of angiogenesis to the inflammatory process in OA is undeniable. New vessels that transport inflammatory cells, nutrients, and oxygen to the site of inflammation can support the inflammatory reaction and thus affect disease progression and pain intensity. Angiogenesis and endothelial damage are the main components of neovascularization and fibrosis, which are quite often observed in patients with COVID-19 and in the post-infectious period. GFs, like cytokines, are critical regulators of tissue damage/repair, and are key components in the pathophysiology of both OA and COVID-19. The mutual ratio of VEGF, FGF-2 and PDGF determines the development and stimulation of angiogenesis. In osteoarthritic joints, angiogenesis is observed in the synovial membrane and is closely related to chronic synovitis and upregulation of HIF-1α. The degree of angiogenesis and inflammation can vary significantly between different patients with OA. VEGF is one of the key pro-angiogenic factors that specifically acts on endothelial cells, promoting vascular permeability that has the ability to induce both physiological and pathological angiogenesis (38). Numerous studies have reported the involvement of VEGF in the progression of degenerative changes in cartilage and indicated its different levels in patients with OA. VEGF expression is found in articular cartilage, osteophytes and hypertrophied chondrocytes, which is regulated by IL-1β, IL-6, TNF-α and HIF-1α (27). Seatan et al. (39) showed that the VEGF level in the synovial fluid of patients with knee OA was ten times higher than the plasma level and correlated with radiographic severity of OA. Because SARS-CoV-2 infection often leads to endothelial dysfunction, VEGF signaling may play a critical role in the pathogenesis of COVID-19. FGF-2, which acts synergistically with VEGF, activates the proliferation and migration of endothelial cells, which regulates the normal development and homeostasis of articular cartilage, in addition, it initiates a macrophage-related angiogenic response during the inflammation. Elevated level of FGF-2 in plasma and synovial fluid of patients with OA was shown, which was correlated with degenerative changes in cartilage and the severity of the disease (40). PDGF is a mitogenic and chemotactic factor for a variety of cells, including chondrocytes, osteoclasts, and osteoblasts, on which it has proliferative effects (41). Therefore, a violation of the signal transduction of the above-mentioned pro-angiogenic factors can contribute to the occurrence and progression of OA. However, unlike cytokines, the levels of most GFs did not increase, but rather decreased with the progression of COVID-19 or during convalescence (42). The decrease in the level of GFs in severe stage of COVID-19 probably occurs due to the depletion of the platelet link of hemostasis, the dysfunction of epithelial, endothelial, and immune cells, which are the main sources of these GFs. The results of studies we have obtained that have reduced levels of VEGF, FGF-2 and PDGF compared to healthy controls expand the recent reports showing differences in circulating levels of several GFs in patients with COVID-19 (43). Some studies indicate dysregulation and elevated levels of these GFs in the blood of patients with COVID-19 (43). It is known that viruses directly or indirectly, involving various mechanisms including HIF-1α, cyclooxygenase 2 (COX-2), NF-kB, IL-6 can increase expression of GFs (44). Therefore, more research is needed to clarify these contradictory results and findings regarding GFs changes in both acute SARS-CoV-2 infection and the post-COVID-19 period. The development of any pathological condition of the organism is usually associated with tissue hypoxia, which affects the activation of intracellular signals, which in turn can contribute to the progression of the disease. Inflammation and hypoxia are constantly present in knee OA and are also the main physiological consequences of SARS-CoV-2 infection, especially in severe cases (45). Violation of oxygen homeostasis in articular cartilage, where its level is usually low, can affect chondrocytes adapted to hypoxic conditions and cause degenerative changes. Pathogenic factors of hypoxia can act at all levels: from systemic to cellular, including factors provoked by hypoxia that can have a cumulative effect on each other. HIF-1α plays a key role in cellular responses to hypoxic conditions. In our research, the results showed no significant changes in the plasma level of HIF-1α in patients with OA, including patients who recovered from COVID-19. Although it has been reported that HIF-1α is found in plasma, it is known to be an unstable protein (has a short half-life, about 5 min), which is rapidly degraded by the enzyme prolylhydroxylase, thus measuring its level requires careful collection and storage conditions for samples (46). The study of Qing, et al. showed an increased concentration and expression of HIF-1α mRNA in the synovial fluid and articular cartilage of patients with primary knee OA when compared to healthy controls, which was significantly correlated with disease severity (47). It is known that under conditions of normoxia the level of cytoplasmic HIF-1α remains low or undetectable due to rapid degradation, while under conditions of hypoxia it stabilizes and accumulates. The stabilized HIF-1α is translocated to the nucleus, where it forms a heterodimer with the corresponding β-subunit of HIF-1, allowing them to modulate the expression of a wide range of genes by binding to DNA sequences, and regulate a number of important protective processes, including angiogenesis, metabolism, cellular proliferation and survival (48). Recent studies have shown that GFs such as insulin-like growth factor (IGF), VEGF, and cytokines such as IL-1 and TNF-α can affect the HIF-1α protein levels and its DNA-binding activity. Regulation of HIF activity can occur through a variety of intracellular signaling pathways, including NF-κB (16). Usually, viral infections are closely related to the stimulation of cellular stress proteins, which is more pronounced in patients with concomitant diseases. The inflammation, hypoxia and angiogenesis additionally burdens the protein folding system. Molecular chaperones in cells work as an integrated network, participating in the folding of newly synthesized polypeptides, refolding of metastable proteins, assembly, regulation of activity, translocation and dissociation of protein complexes, and degradation of misfolded proteins. In addition to their chaperone functions, they also play an important role in cell signal transduction and apoptosis regulation. There is an assumption that the HSPs expression profiles can act as biomarkers of the severity of some disease (49). It was shown that the concentration of HSP60 in plasma was positively correlated with acute lung injury and systemic inflammatory response in patients with severe extrapulmonary lesions (50). However, data on the biological functions of these proteins in SARS-CoV-2 infection are currently limited. In the study of HSPs in the pathogenesis of OA, the anabolic effect of HSP60 in the joints was indicated and a decrease in its concentration and mRNA level in the cartilage tissue and synovial fluid of patients with OA was found (51). These results of the research were consistent with our data on decreased plasma HSP60 levels in patients with OA, as well as those who recovered from COVID-19. It is known that the ability of HSP60 to respond to various stresses largely depends on changes in its localization inside the cell; protein secretion can be carried out in response to the activation of pro-inflammatory cytokines such as IL-1β and TNF-α. On the other side, circulating HSP60 is recognized as a powerful inducer of the release of pro-inflammatory mediators by different cells (52). Our results did not reveal any significant changes in the level of HSP70 in the blood of patients with OA, as well as those who recovered from COVID-19. In studies (53) it was shown that the level of HSP70 in plasma and synovial fluid was directly correlated with the severity of OA, while the protein level in synovial fluid was three times higher than in the blood, which was associated with local inflammation in the joint. Similar results are presented by Schett et al. (54) showed an elevated of HSP70 level in the synovium of patients with rheumatoid arthritis (RA), but its level did not change in patients with OA. It was found that the expression of HSP70 genes was mainly increased in the early stage of OA, which was accompanied by an increase in protein concentration and mRNA expression (53). It has been shown that HSP70 can limit NF-ĸB activation and proteasomal degradation of their inhibitory subunits IĸB α. Thus, by suppressing inflammation and preventing oxidative stress, HSP70 may indirectly regulate COVID-19 (49, 55). A possible connection between the dysregulation of stress proteins and the severity of SARS-CoV-2 infection can be assumed, but this still requires experimental confirmation. CONCLUSION Pro-inflammatory cytokines, which play a crucial role in the catabolism of articular cartilage and pro-angiogenic factors VEGF, FGF-2 and PDGF, regulator of the hypoxic response HIF-1α and heat shock proteins HSP60 and HSP70 providing support for cellular homeostasis, were included in the study because of their importance in the pathogenesis of both OA and COVID-19. Despite a long period of time from acute SARS-CoV-2 infection, systemic deregulation of the cytokine profile was established in patients with OA, which could indicate chronic or residual inflammation in the organism. The long post-COVID-19 period was characterized by a significant reduction in levels of pro-angiogenic GFs, most pronounced in cases of VEGF and PDGF in the plasma of patients with OA. It can be assumed that there are mechanisms that increase or decrease the regulation of GFs at different stages of the disease, including after infection. Against the background of a decrease in the levels of the angiogenic parameters in patients with joint pathology in conditions of both the presence and absence of a history of SARS-CoV-2 infection, this analysis did not reveal any significant difference in the plasma level of transcription factor HIF-1α. A decrease in the level of stress protein HSP60 in the blood of patients with OA knee joints, as well as those patients who have undergone SARS-CoV-2 infection, has been established. We are aware that the sample used in the presented study was small, and that a repetition with a larger sample size will be needed to confirm the results obtained that may cause controversy. Nevertheless, the established results in combination with literature data suggest the potential role of pro-inflammatory and angiogenesis-related factors as prognostic biomarkers of OA progression, as well as distinguishing between different inflammatory conditions related to the musculoskeletal system or the development of post-COVID complications.

## Data availability statement

The original contributions presented in the study are included in the article/Supplementary material, further inquiries can be directed to the corresponding author.

## Ethics statement

The studies involving human participants were reviewed and approved by Ethics Committee at the ESC “Institute of Biology and Medicine,” Kyiv, Ukraine (the protocol № 3, 12.04.2022). The patients/participants provided their written informed consent to participate in this study.

## Author contributions

DK, KS, and MK conceived and designed analysis, collected the data. OT and LO wrote the manuscript. TV, TH, LK, NR, OS, and TF revised and edited the manuscript. All authors contributed to the article and approved the submitted version.

## Conflict of interest

The authors declare that the research was conducted in the absence of any commercial or financial relationships that could be construed as a potential conflict of interest.

## Publisher’s note

All claims expressed in this article are solely those of the authors and do not necessarily represent those of their affiliated organizations, or those of the publisher, the editors and the reviewers. Any product that may be evaluated in this article, or claim that may be made by its manufacturer, is not guaranteed or endorsed by the publisher.
