# Supplementary figures and images for: Circulating levels of inflammatory cytokines and angiogenesis-related growth factors in patients with osteoarthritis after COVID-19
Source: Front Med (Lausanne). 2023 Jul 6;10:1168487. doi: 10.3389/fmed.2023.1168487 (PMC10358362; doi:10.3389/fmed.2023.1168487)

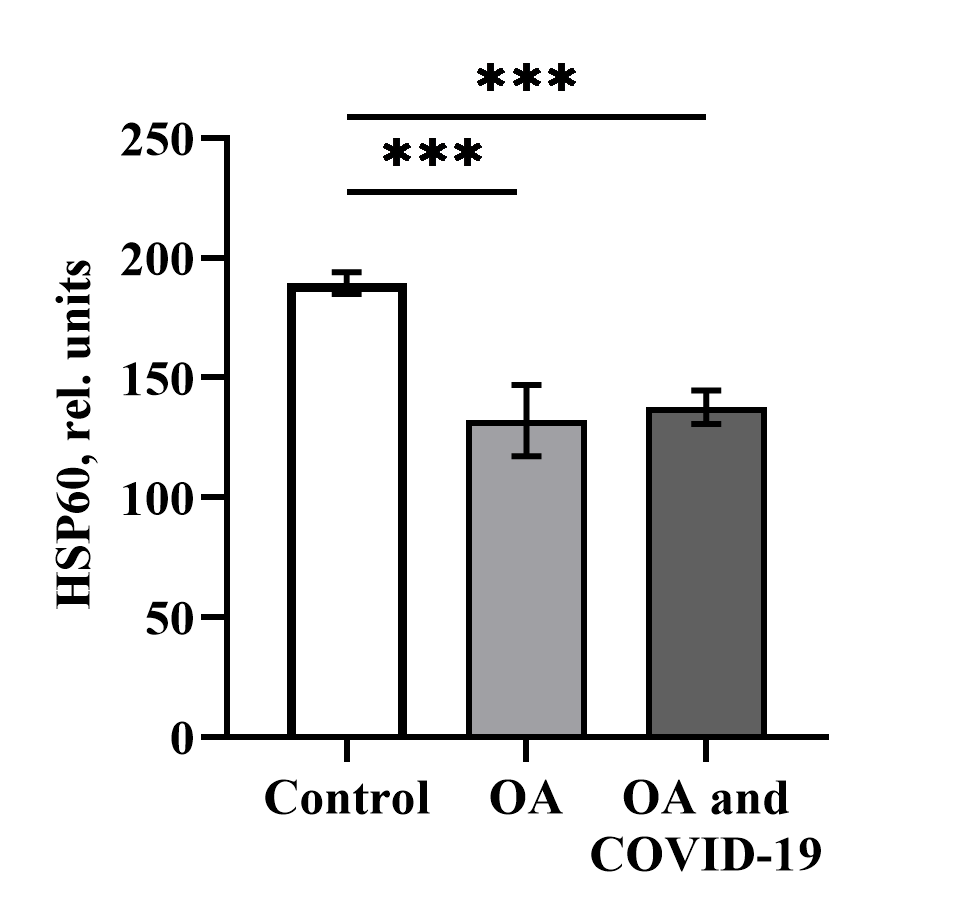

Supplement: Supplementary file 1 [file Image_1.PNG]

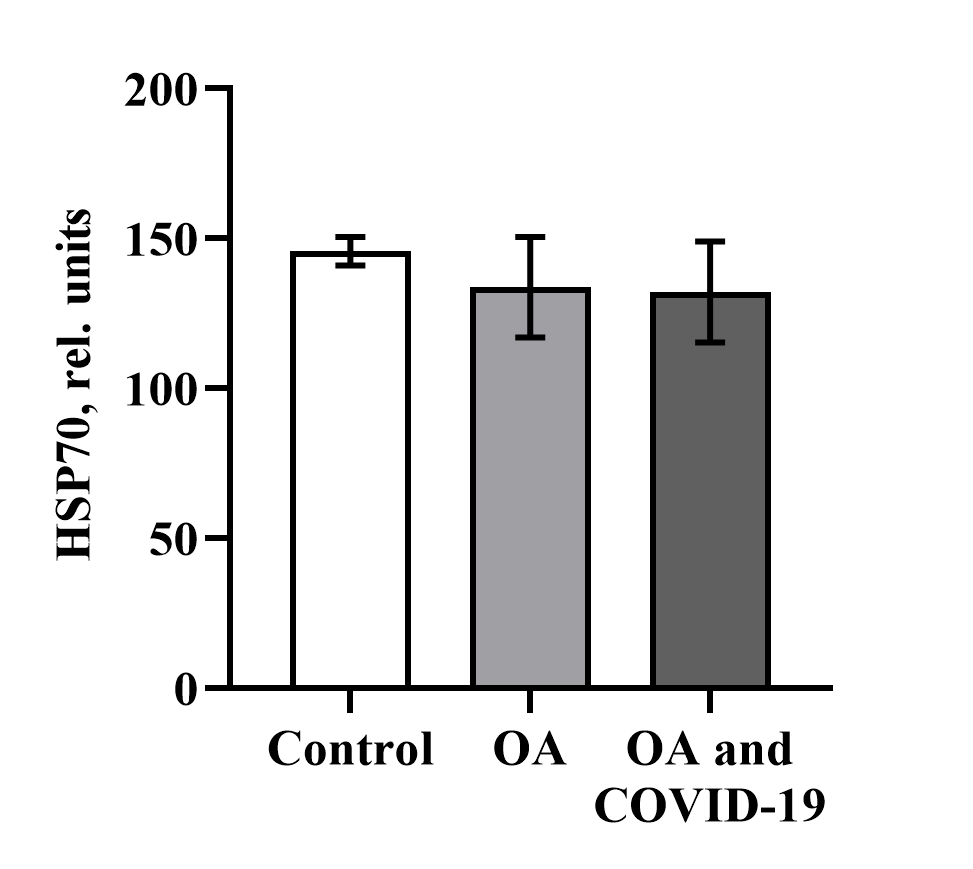

Supplement: Supplementary file 2 [file Image_2.PNG]
